# Limitations of the UNAIDS 90-90-90 metrics: a simulation-based comparison of cross-sectional and longitudinal metrics for the HIV care continuum

**DOI:** 10.1097/QAD.0000000000002502

**Published:** 2020-02-14

**Authors:** Noah A. Haber, Catherine R. Lesko, Matthew P. Fox, Kimberly A. Powers, Guy Harling, Jessie K. Edwards, Joshua A. Salomon, Sheri A. Lippman, Jacob Bor, Angela Y. Chang, Andrew Anglemyer, Audrey Pettifor

**Affiliations:** a Carolina Population Center, University of North Carolina at Chapel Hill, Chapel Hill, North Carolina; b Meta-Research Innovation Center at Stanford University, Stanford, California; c Department of Epidemiology, Johns Hopkins University, Baltimore, Maryland; d Department of Global Health; e Department of Epidemiology, Boston University School of Public Health, Boston, Massachusetts, USA; f Department of Internal Medicine, Faculty of Health Sciences, Health Economics and Epidemiology Research Office, School of Clinical Medicine, University of the Witwatersrand, Johannesburg, South Africa; g Department of Epidemiology, University of North Carolina at Chapel Hill, Chapel Hill, North Carolina, USA; h Institute for Global Health, University College London, London, UK; i Africa Health Research Institute, Durban, KwaZulu-Natal; j MRC/Wits Rural Public Health and Health Transitions Research Unit (Agincourt), School of Public Health, Faculty of Health Sciences, University of the Witwatersrand, Johannesburg, South Africa; k Harvard Center for Population and Development Studies, Cambridge; l Department of Epidemiology, Harvard T.H. Chan School of Public Health, Boston, Massachusetts; m Center for Health Policy and Center for Primary Care and Outcomes Research, Department of Medicine, School of Medicine, Stanford University, Stanford; n Department of Medicine, Division of Prevention Science, University of California San Francisco, San Francisco, California; o Department of Global Health and Population, Harvard T.H. Chan School of Public Health, Boston, Massachusetts, USA; p Danish Institute for Advanced Study, Copenhagen, Denmark; q Naval Postgraduate School, Operations Research Department, Monterey, California, USA; r Department of Preventive and Social Medicine, University of Otago, Dunedin, New Zealand.

**Keywords:** cascade, continuum, cross-sectional, longitudinal, Joint United Nations Programme on HIV/AIDS (UNAIDS) 90–90–90

## Abstract

The Joint United Nations Programme on HIV/AIDS (UNAIDS) 90–90–90 and other cross-sectional metrics can lead to potentially counterintuitive conclusions when used to evaluate health systems’ performance. This study demonstrates how time and population dynamics impact UNAIDS 90–90–90 metrics in comparison with a longitudinal analogue.

**Design:**

A simplified simulation representing a hypothetical population was used to estimate and compare inference from UNAIDS 90–90–90 metrics and longitudinal metrics based on Kaplan–Meier-estimated 2-year probability of transition between stages.

**Methods:**

We simulated a large cohort over 15 years. Everyone started out at risk for HIV, and then transitioned through the HIV care continuum based on fixed daily probabilities of acquiring HIV, learning status, entering care, initiating antiretroviral therapy (ART), and becoming virally suppressed, or dying. We varied the probability of ART initiation over three five-year periods (low, high, and low). We repeated the simulation with an increased probability of death.

**Results:**

The cross-sectional probability of being on ART among persons who were diagnosed responded relatively slowly to changes in the rate of ART initiation. Increases in ART initiation rates caused apparent declines in the cross-sectional probability of being virally suppressed among persons who had initiated ART, despite no changes in the rate of viral suppression. In some cases, higher mortality resulted in the cross-sectional metrics implying improved healthcare system performance. The longitudinal continuum was robust to these issues.

**Conclusion:**

The UNAIDS 90–90–90 care continuum may lead to incorrect inference when used to evaluate health systems performance. We recommend that evaluation of HIV care delivery include longitudinal care continuum metrics wherever possible.

## Introduction

The treatment continuum or cascade of care divides the process by which individuals and populations progress from HIV acquisition through ongoing viral suppression into discrete steps [1]. Cascades are typically represented as the number or proportion of people in a population at a given cross-section in time. The Joint United Nations Programme on HIV/AIDS (UNAIDS) 90–90–90 targets are ubiquitous continuum of care goals for monitoring and evaluating the global HIV response. Achieving high or low proportions in any target is generally attributed to successes or gaps in healthcare system specific to transitioning from the denominator state to the numerator [2]. The targets state that 90% of all people living with HIV should know their HIV status, 90% of all people with diagnosed HIV infection should receive sustained antiretroviral therapy (ART), and 90% of all people receiving ART should have viral suppression in order to end the AIDS epidemic [3].

However, because they rely on a cross-sectional framework to describe dynamic, longitudinal, and interrelated processes, the UNAIDS 90–90–90 metrics may yield misleading inference about health systems performance [4,5]. Health systems changes can have counterintuitive impacts on cross-sectional performance metrics, potentially resulting in misinterpretation, poor policy and resource allocation decisions, and adverse mortality and morbidity outcomes.

Longitudinal continuums have been emerging as an approach to address these issues, which have been used to evaluate heath systems performance in a variety of settings and formulations [1,6–15]. While cross-sectional continuums yield a snapshot of the current state of a system, longitudinal continuums describe movement between states over time, conceptually analogous to prevalence and incidence, respectively. One longitudinal cascade approach is the ‘HIV testing and treatment cascade,’ [16] which measures person time from the one common entry event, such as HIV infection or clinical diagnosis, to all subsequent stages [10,17]. A second approach uses a stage-by-stage method, where each stage depends on completion of the previous, analogous to the UNAIDS 90–90–90 targets [4]. In the current article, we focus on the latter approach to conduct a side-by-side comparison of the UNAIDS 90–90–90 metrics against a longitudinal analogue.

The mechanisms by which the UNAIDS 90–90–90 and other cross-sectional metrics may yield misleading inference about health systems performance – and how longitudinal approaches may resolve these issues – are largely undocumented and poorly understood. We focus on four issues with the UNAIDS 90–90–90 metrics: they are relatively slow to respond to emerging conditions; they can change even when there is no underlying change in system performance; they are subject to unintuitive and complicated between-stage interactions; and they are counter intuitively impacted by mortality.

We designed this study to examine and demonstrate how summarizing a longitudinal, interrelated set of dynamic processes using cross-sectional proportions can impact the UNAIDS 90–90–90 metrics in unintuitive ways. We also demonstrate how longitudinal approaches may provide more direct, robust, and reliable metrics for evaluating HIV systems performance.

## Methods

We developed a stochastic, individual-based simulation model to represent a stylized introduction and expansion of ART, changing only the ART initiation rate over time. We simulate two scenarios: one with relatively low mortality rates (baseline) and one with relatively high mortality rates (5× the baseline) sustained over the entire simulated 15-year period. We compared cross-sectional and longitudinal continuum metrics both within and between the simulated periods and scenarios to assess their responses to the changing ART and mortality conditions.

### Simulation structure and baseline parameters

The simulation was based on a Markov chain process, modelled after the ‘HIV States and Transitions Framework’ [18]. The model classifies each individual into one of the following ordinal states at each point in time: at risk for HIV, living with HIV, knows status, in care, on ART, virally suppressed, or dead. The simulations begin with every person being at risk for HIV. At daily time steps, each simulated person in the model may remain in the same state, transition to the next state, or die, according to defined probabilities that are evaluated stochastically for each person at each time step. Transition rates are assumed to be identical for all persons for any given transition. For simplicity, the model did not include any skipping of stages, ‘side door’ [19] entry into the continuum, reversion to previous stages, churning statuses [20], or viral suppression without treatment [21,22].

**Fig. 1:**
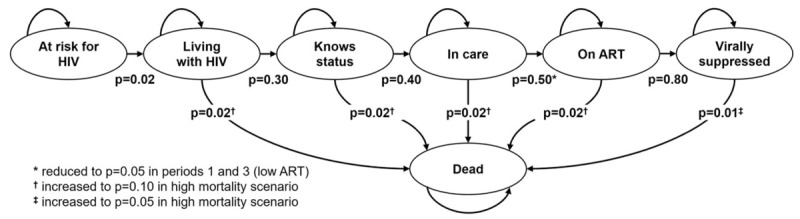
Diagram of Markov states and transitions.

Figure 1 summarizes the model states and transitions, including the annual probabilities used in the simulations. The simulation structure and parameters used in this simulation were generated and selected primarily to aid conceptual and visual clarity for the demonstration purposes. While we believe them to be broadly plausible, they do not necessarily represent any specific populations, country, or time period.

Pictured transition probabilities are annual transition probabilities for each Markov state, and were converted to daily probabilities using the formula: .

### Scenarios

We generated two separate simulation scenarios: a baseline (relatively low) and a high mortality scenario. The baseline mortality scenario was designed to demonstrate how the main continuum metrics respond to conditions even without substantial mortality, while the high mortality scenario demonstrates the additional complications that occur with increased mortality when people are removed from cross-sectional metrics due to death, a competing event.

Each scenario had three periods, each 5 years long: a low ART period, a normal ART period, and a second low ART period. In Period 1, the ART initiation rate (0.05 per year) was set to be 1/10th the baseline rate. In Period 2, we increased the ART initiation rate (0.50 per year) to represent introduction of wide scale ART availability. In Period 3, we returned to the lower ART initiation rate (0.05 per year). This last period may conceptually represent a supply shortage or reversion to prior policies. In all periods, mortality rates before viral suppression were twice that as after viral suppression.

Additional model runs with slightly altered parameters were generated on an ad-hoc basis to test the sensitivity of model outcomes to parameter changes, including modifying the mortality rate, viral suppression rate, and period lengths.

### Continuum metrics

For each scenario we compared two sets of different continuum metrics from the simulated data first, cross-sectional continuum metrics based on the UNAIDS 90–90–90 targets; and second, stage-by-stage longitudinal continuum metrics analogous to the UNAIDS targets. Stage entry and completion events are the events that define the denominator and numerator of a given metric, respectively.

### UNAIDS 90–90–90 metrics

The UNAIDS 90–90–90 metrics were based on cross-sectional proportions. The denominator for each of the three stages was the number of people who were alive and had completed the entry event by the measurement date, and the numerator was the number of people who were alive and had completed both the stage entry and stage completion event by the measurement date. This yielded three probabilities on each date: the proportion of people who knew their status out of those who were living with HIV; the proportion of people who had initiated ART out of those who had been diagnosed and were alive; and the proportion of people who were virally suppressed out of those who had initiated ART and were alive.

### Longitudinal 90–90–90 metrics

The longitudinal 90–90–90 metrics measured the flow of people as they passed between stages. We used Kaplan–Meier curves [23] to estimate the cumulative incidence of a stage completion event, where day 0 was the day on which the stage entry event occurred. Individuals who had not transitioned into a subsequent stage by the last date of the measurement period were right censored on that date. Death was considered a failure to achieve the completion event (a competing event) rather than a censoring event. As such, people who had died remained in the ‘at-risk’ population to reflect that they died before transitioning, avoiding the issue of death inflating the probability of transitioning to subsequent stages. This approach is effectively equivalent to the Aalen–Johnson cumulative incidence function in the presence of the competing risk of death, given that they are strictly hierarchical and censoring occurs for both potential risks at the same time [24,25].

Estimates for each stage were restricted to persons who completed the stage entry event within 2 years prior to the measurement date; thus, the time-horizon of measurement was up to 2 years from the stage entry event to the stage exit event. For example, the longitudinal continuum metrics for the transition between knowing status and initiating ART at the end of year 5 would consider only those individuals who learned their status for the first time in years 4 or 5, include only ART initiation events occurring before the end of year 5, and would censor individuals who were alive but had not yet initiated ART by the end of year 5. As a result, the longitudinal continuum metrics in this analysis do not reflect events that occur more than 2 years from the stage entry event.

### Analysis

The state of each cascade was measured and given at the last day of each simulated year. For the UNAIDS 90–90–90 metrics, this was the cross-sectional status of the continuum, or a repeated cross-section similar to the approach of Nosyk *et al.*[26]. For the longitudinal metrics, these were the cumulative incidence curves for each of the three transitions from the 2 years prior to the last day of each simulated year.

All analyses were performed in R v3.6.1 [27]. The longitudinal continuum metrics were generated using the longitudinalcascade package, v0.3.2.1 [28], which was developed by the first author for public use prior to this simulation study. All code and simulated data are included as supplements to this article.

## Results

Total population proportions from the simulation are shown in Appendix 1 and Appendix 2, with daily continuum comparisons available in a video supplement.

### Standard mortality scenario

#### UNAIDS 90–90–90 metrics

**Fig. 2:**
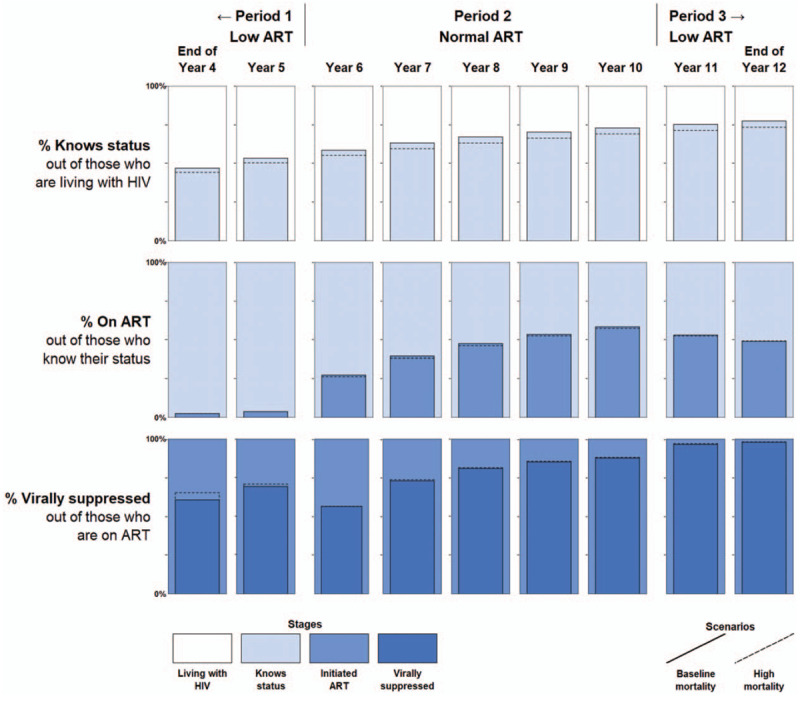
UNAIDS 90–90–90 metrics.

The cross-sectional UNAIDS 90–90–90 metrics are shown as a series of annual, end-of-year cross-sections for years 4–12 of the simulation in Fig. 2, with numerical values in Appendix 3.

This chart shows the results of the cross-sectional UNAIDS 90–90–90 metrics at the end of years 4–12 of the simulation. Each row represents one of the three UNAIDS 90–90–90 metrics, where the background colour indicates the stage for the denominator, and the bars represent the metric. The main bars (demarcated by solid lines) represent the normal mortality scenario, while the dotted lines represent the results for the high mortality scenario. The areas in the high mortality scenario are left transparent to increase readability and comparison with the low mortality scenario.

The top row shows the first of the three UNAIDS 90–90–90 metrics: the percentage of persons living with HIV who know their status out of those who are alive and living with HIV. This proportion rose slowly over time in all periods, reflecting the steady accumulation of HIV diagnoses via the constant HIV diagnosis rate against a background where the mortality rate was lower than the HIV incidence rate.

The proportion of those who are on ART of those who know their status was slow to respond to the changes in ART initiation probabilities between model periods, as seen in the second row of Fig. 2. At the end of year 5, only 4% of people who knew their status were on ART, reflecting the very low probability of any diagnosed individual initiating ART. Once ART rates were raised to ‘normal’ levels, the proportion who were on ART jumped to 27% at the end of year 6, reflecting rapid uptake from the backlog of individuals built up by this point who knew their status but had not yet initiated ART. The proportion of individuals who had initiated ART increased steadily to 58% at the end of year 10, but then decreased only slowly in Period 3 despite the rapid drop in ART initiation probabilities at the start of year 11.

The proportion of people who have become virally suppressed out of those who have initiated ART appears in the bottom row of Fig. 2. The initial dip in the proportion who have achieved viral suppression in year 6, when ART roll-out began, was largely caused by improving conditions in other periods; as more people initiated ART, the denominator grew, and it took time for the numerator to catch up. Despite no change in the viral suppression rate after ART initiation, the dip in year 6 leads to the impression that viral suppression was a substantial bottleneck in the continuum at that time. That impression could lead to erroneous conclusions, such as ‘the people being reached with expanded ART access have worse adherence than those who were initiated ART in prior periods’ or ‘adherence counselling should be prioritized for funding.’ As Period 2 continued, the proportion of people who were virally suppressed continued to increase as this initial influx people who had initiated ART subsequently achieved viral suppression, again despite no actual changes in the viral suppression rate among those who are on ART.

**Fig. 3:**
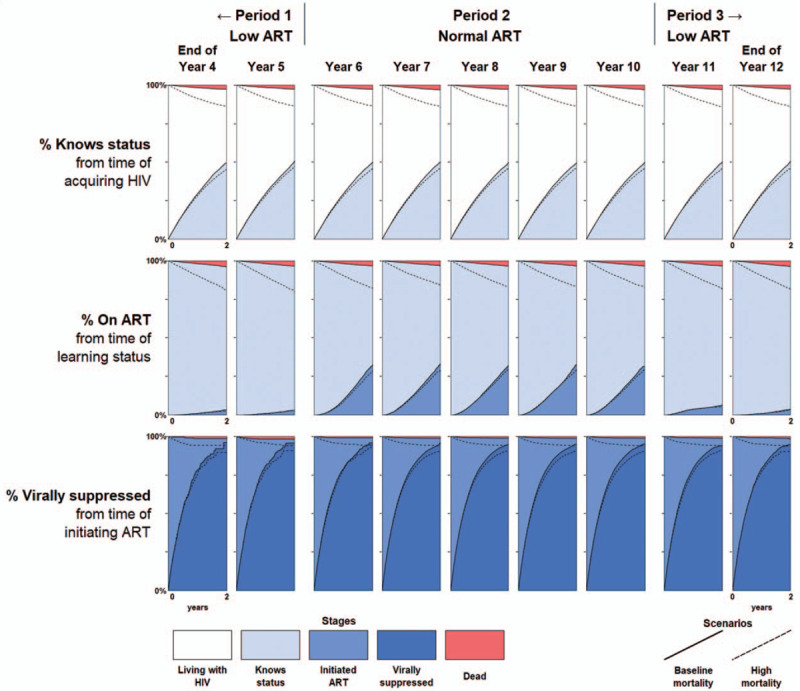
Longitudinal 90–90–90 metrics.

The comparable chart for the longitudinal 90–90–90 metrics is shown in Fig. 3. Each row reflects the cumulative incidence function for the transition from one stage to the next over the prior 2 years, rather than the total accumulation of people who have reached each stage to date. Because the cumulative incidence function is determined almost exclusively by the transition probabilities corresponding to each stage, the first and last rows of the longitudinal 90–90–90 continuum remain virtually unchanged over time, reflecting the fact that the underlying rates of HIV acquisition, learning status, and viral suppression remained constant throughout the simulation.

Longitudinal 90–90–90 metrics calculated at the end of years 4–12 of the simulation. Each row represents one of the three cumulative incidence curves in the longitudinal continuum, where the background colour indicates the stage for the denominator. The main filled curves (demarcated by the solid lines) represent the normal mortality scenario, while the dotted lines represent the results for the high mortality scenario. The areas corresponding to differences between the low and high mortality scenarios are left transparent for readability and comparison with the low mortality scenario.

Just as the top and bottom rows of Fig. 3 properly reflect the constant transition probabilities underlying their corresponding metrics, the middle row properly captures the changing probability of ART initiation across calendar periods. The response to the changing ART initiation probabilities was nearly immediate. This feature of the longitudinal continuum is largely due to its explicit consideration of only recent transitions from stage to stage, as opposed to a cross-sectional metric that reflects prevalence arising from transitions that had occurred at any point in the past.

The longitudinal metrics also yield some additional insight in the form of person-time. The cross-sectional metrics showed relatively high prevalence of being on ART and being virally suppressed at the end of Period 2, seemingly suggesting relatively high rates of ART initiation and viral suppression. These cross-sectional metrics would have continued to increase had the ‘normal ART’ conditions persisted, reaching the full UNAIDS 90–90–90 targets despite no improvements in health systems performance after the switch to higher ART initiation probabilities at the start of year 6. However, the longitudinal metrics reveal a large amount of person-time between those transitions, reflecting the time in which individuals remained virally unsuppressed and highlighting the remaining gaps for intervention.

#### High mortality scenario

Increasing mortality had a complex and potentially counterintuitive impact on the health systems performance implied by the UNAIDS 90–90–90 metrics, as shown in the dotted lines in Fig. 2. The UNAIDS 90–90–90 metrics were largely unresponsive to the five-fold increase in the annual mortality rate. In the case of the first and second components, we observe implied overall decreases in health systems performance. However, the third component appears to imply *improved* health systems performance with higher mortality. The reason for this observed increase in viral suppression is that a higher proportion of people were dying and being removed from the denominator as compared with the low mortality scenario. Additional sensitivity tests show that this effect is exacerbated when the mortality rate is increased and/or when the viral suppression rate is reduced.

The longitudinal 90–90–90 metrics resulted in unambiguous decline in the measurements of health systems performance of all stages when mortality increased, as shown in Fig. 3. The proportion of people who had died before transitioning into the next stage is shown in the red area at the top of each panel, which is visually larger for all previral suppression stages in the continuum under the high mortality scenario. Furthermore, higher mortality also results in an unambiguous reduction in the proportion of people transitioning into the next stage for all three metrics. The nonambiguity of mortality in the longitudinal 90–90–90 metrics is due to people remaining in the denominator even after dying, as death was treated as a failure to achieve an even through the competing risk of death rather than failure to observe an event (censoring).

## Discussion

Metrics used to evaluate health systems performance should be highly responsive to and reflective of current conditions, independently identify performance between stages, and avoid implying higher performance in scenarios with poorer outcomes. The longitudinal continuum metrics perform better than the UNAIDS 90–90–90 metrics on all three of these criteria in our simulation. The cross-sectional UNAIDS 90–90–90 metrics were relatively slow to respond to changes, reflected changes in stage transition rates other than the primary stage transition event under study, and implied improvements in health systems performance under higher mortality rates. By contrast, the longitudinal continuum was quick to respond, maintained relative independence in reflecting stage transitions, and unambiguously indicated lower performance with higher mortality.

Although we used a simplistic model, more realistic conditions would not resolve the issues with the UNAIDS 90–90–90 illustrated here. Perhaps the least realistic aspect of our simulation was having discrete periods with sharp discontinuities instead of gradual changes over time. A more realistic simulation could incorporate demographics, viral load, disease stage, and different propensities for moving through the continuum according to these factors. Further, the longitudinal continuum model itself could be made more complex with skipped and out-of-order transitions, ‘churn’ in and out of states of care retention and viral suppression [10,20,29], and/or ‘side doors’ [19]. While these nuances are important to include and integrate into performance metrics in real-world settings, we have no reason to believe that simulating them would have any notable impact on the qualitative conclusions from this study.

The simplicity of implementing the UNAIDS 90–90–90 metrics belies their complex and potentially misleading interpretations when used as health systems performance indicators. Describing the current status of the HIV care continuum with cross-sectional measures can obscure how past periods impacted the current cross-section. Repeated cross-sectional data over time can help improve inference by hinting at transition rate changes, but still necessitates sophisticated understanding and modelling of population dynamics, as seen in comparing cross-sectional and longitudinal metrics using real world data over the ART roll out in KwaZulu-Natal, South Africa [4].

While the longitudinal metrics proposed here are better able to evaluate current systems performance by directly reflecting transition times, they also have tradeoffs. First, they require large and sustained investment in data collection, cleaning, and analysis efforts which are difficult to achieve at scale [30,31] and may be limited to particular geographic regions. In the case of the first metric, determining the date of the stage HIV acquisition, data collection would require frequent repeated testing of a representative population. Second, longitudinal metrics require more complicated analysis and interpretation because they include time. This potential limitation can be mitigated or eliminated by choosing easily interpretable metrics, as discussed below, and/or standardizing analysis methods. Third, longitudinal metrics do not necessarily capture all relevant time horizons. In the example we present in this article, the 2-year data collection and time horizon inherently ignores persons whose transition times exceed longitudinal time horizons. This issue can be mitigated by extending the stage eligibility period, at the cost of the results being less specific to recent conditions.

Target setting using the longitudinal continuum metrics can be as simple as adding a ‘within X time’ component to existing percentage goals, provided appropriate time thresholds [32]. More comprehensive targets could utilize area under the survival curve, or restricted mean survival time [33] metrics. Alternatively, targets can be measured along the reverse axis and measure median or other percentile time to transition, noting that these percentiles may never be reached in many cases. Longitudinal continuum metrics may also include loss of person-time due to churning states, depending on how they are defined.

We recommend utilizing a hybrid of longitudinal and cross-sectional continuum metrics for health systems evaluation and target setting. Setting precise definitions and presentation style for international agendas requires coordination between a diverse set of stakeholders, but a plausible example set of targets could leverage the best and most practical aspects of both. The first two metrics might be cross-sectional targets based on the population living with HIV: 90% of people who are living with HIV should be diagnosed and engaged with care; and at least 73% of people who are living with HIV should be virally suppressed. Although these two targets are likely to be relatively unresponsive in real time, they do not run the risk of issues related to between-stage denominator interactions over time that impact current UNAIDS 90–90–90 metrics. The second target collapses the original 90–90–90 to directly assess its original purpose: keeping viral suppression sufficiently high to dampen spread of HIV. In addition, two longitudinal targets could replace the latter two targets in the current UNAIDS 90–90–90. In keeping with the ‘90–90–90’ theme: 90% of people who are newly linked to care should be on ART within 90 days of linking to care, and 90% of people who are newly on ART should be virally suppressed within 90 days of starting ART. These two longitudinal measures allow stage-specific assessments of health systems performance.

We strongly recommend that existing cross-sectional continuum measurements be viewed with a critical understanding of their limitations, and that longitudinal metrics be incorporated in evaluation of progress and decision-making around HIV policies and strategies wherever possible. We further recommend that major funders invest in systematic data collection efforts to allow longitudinal analysis and calculation of longitudinal care continuum metrics, including expanding existing regional cohort data, introducing new longitudinal HIV monitoring efforts, coordinating across existing monitoring efforts, and expanding and incorporating clinical data networks [34].

## Acknowledgements

No funding was provided specifically for this work: N.A.H. was supported by CPC NICHD-NRSA Population Research Training Grant T32 HD007168. J.B. was supported under grants 1K01MH105320 and 1R01HD084233. J.K.E. was supported under NIH K01AI125087. G.H. was supported by a fellowship from the Wellcome Trust and Royal Society (210479/Z/18/Z). C.R.L. was supported by K01 AA028193.

All authors contributed substantively to this study and article: Specifically, authors filled the following roles: Study conception: N.A.H., A.P.; Study design: N.A.H., C.R.L., M.P.F., K.A.P., G.H., J.K.E., J.A.S., A.P.; Data collection and execution of study design: N.A.H.; article writing: N.A.H., C.R.L., M.P.F., K.A.P., J.A.S. article revisions: N.A.H., C.R.L., M.P.F., K.A.P., G.H., J.K.E., J.A.S., S.A.L., J.B., A.Y.C., A.A., A.P.

### Conflicts of interest

There are no conflicts of interest.

## Supplementary Material

Supplemental Digital Content

## Supplementary Material

Supplemental Digital Content
