# Supplementary material for: Limitations of the UNAIDS 90-90-90 metrics: a simulation-based comparison of cross-sectional and longitudinal metrics for the HIV care continuum
Source: AIDS. Author manuscript; Available in PMC 2020 Jun 23. (PMC7253182; doi:10.1097/QAD.0000000000002502)
Supplement: Appendix tables and figures [file NIHMS1597547-supplement-Appendix_tables_and_figures.pdf]

Appendix

Appendix 1: Continuum states over time by repeated cross-section, normal mortality scenario

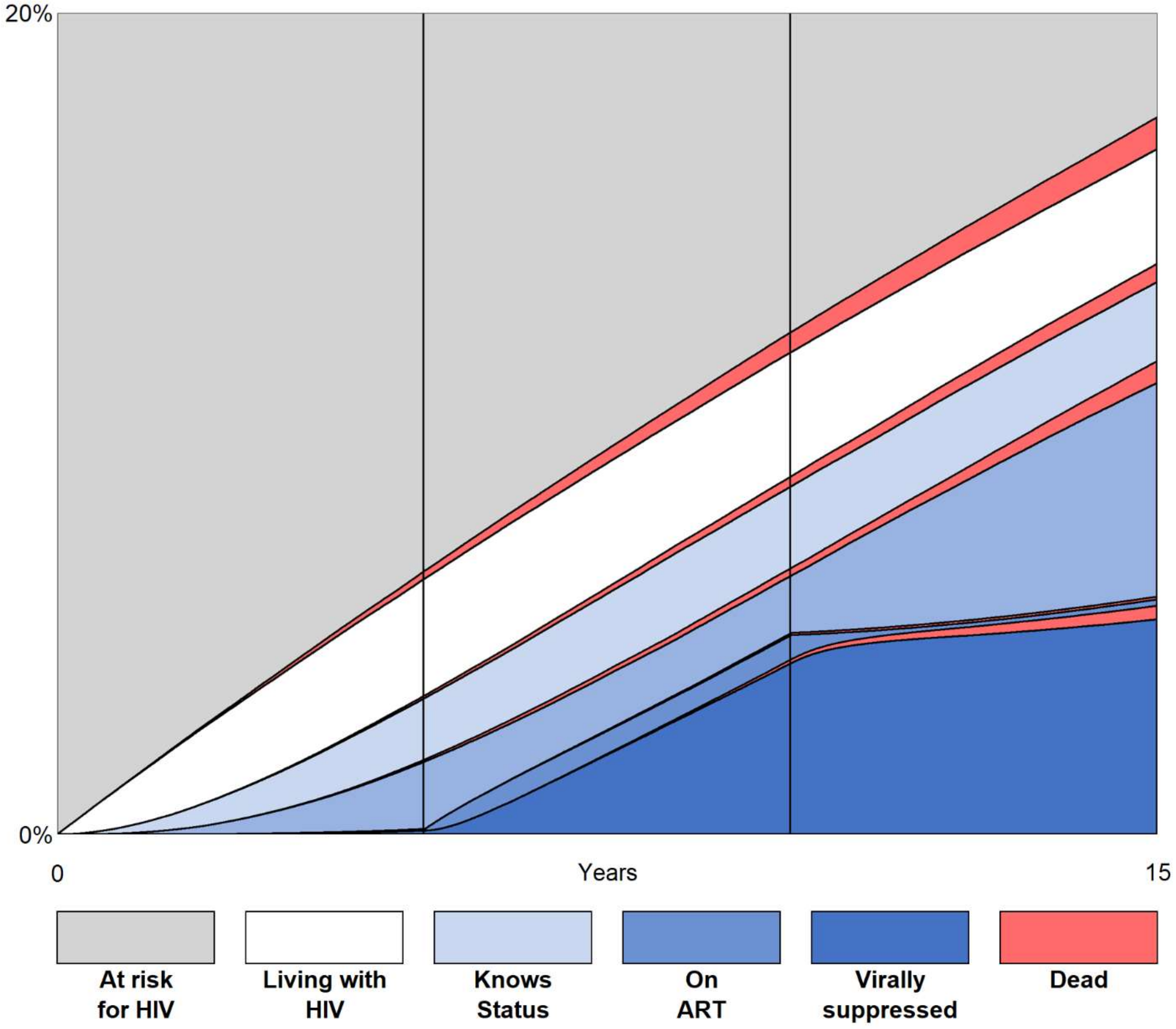

The stacked area represents the proportion of people in a given state on each day in the simulated scenario. All persons in a given state had passed through all prior states (e.g. persons who are in the “On ART” area had already passed through being at risk for HIV, living with HIV, and Knows status). The death state is shown in the state in which a person was when they died. The vertical bars represent the three simulated periods.

Appendix 2: Continuum states over time by repeated cross-section, high mortality scenario

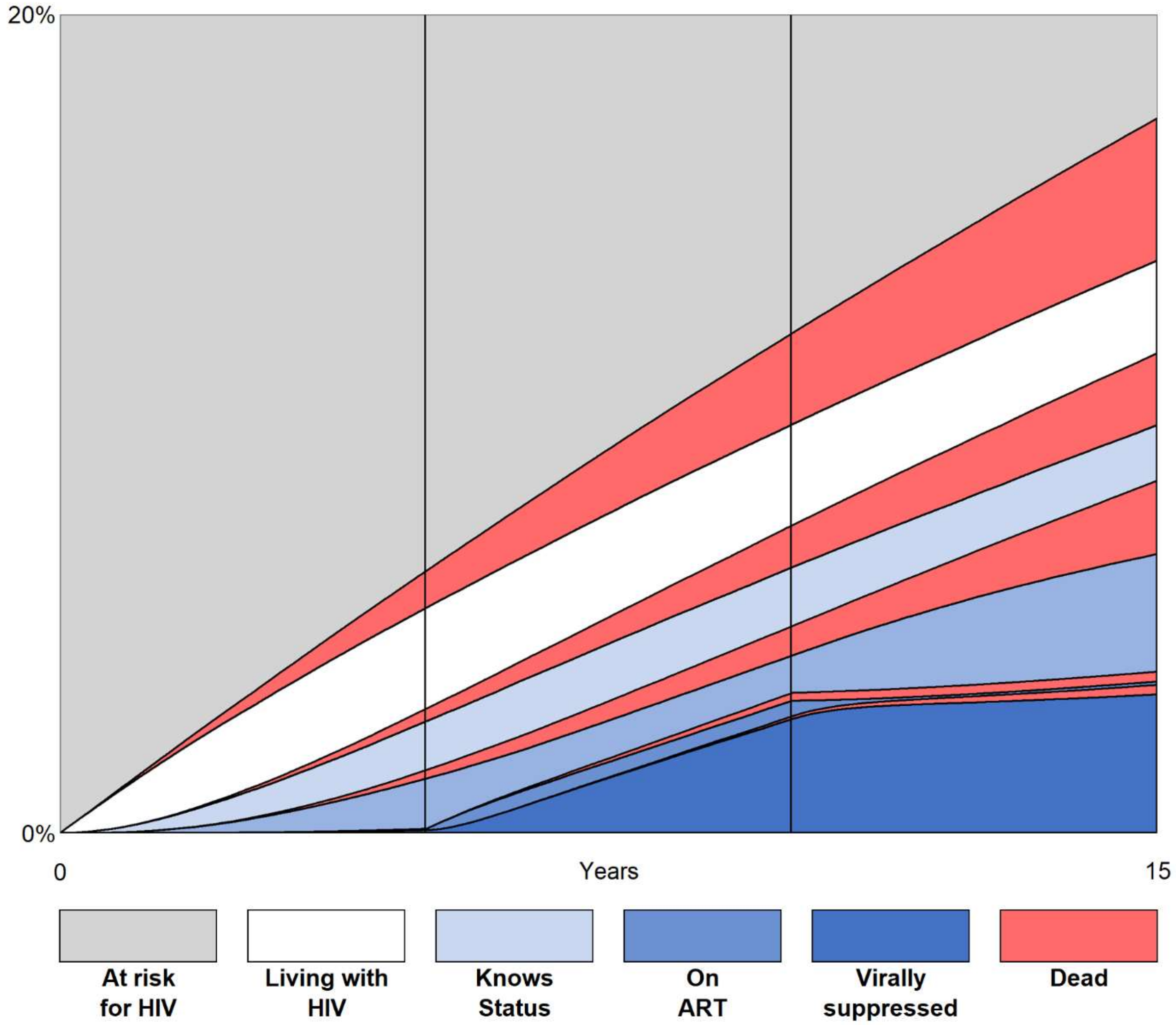

The stacked area represents the proportion of people in a given state on each day in the simulated scenario. All persons in a given state had passed through all prior states (e.g. persons who are in the “On ART” area had already passed through being at risk for HIV, living with HIV, and knows status). The death state is shown in the state in which a person was when they died. The vertical bars represent the three simulated periods.

Appendix 3: UNAIDS 90-90-90 metrics

	Years after simulation start	Baseline mortality scenario			High mortality scenario		
		% Knows status out of those who are living with HIV	% On ART out of those who know their status	% Virally suppressed out of those who are on ART	% Knows status out of those who are living with HIV	% On ART out of those who know their status	% Virally suppressed out of those who are on ART
Low ART (Period 1)	1	16%	0%	33%	16%	0%	33%
	2	29%	1%	44%	29%	1%	44%
	3	39%	2%	49%	39%	2%	49%
	4	47%	3%	61%	47%	3%	61%
	5	53%	4%	69%	53%	4%	69%
Normal ART (Period 2)	6	59%	27%	56%	59%	27%	56%
	7	63%	40%	73%	63%	40%	73%
	8	67%	48%	81%	67%	48%	81%
	9	70%	53%	85%	70%	53%	85%
	10	73%	58%	88%	73%	58%	88%
Low ART (Period 3)	11	75%	53%	96%	75%	53%	96%
	12	77%	49%	98%	77%	49%	98%
	13	79%	46%	98%	79%	46%	98%
	14	81%	44%	98%	81%	44%	98%
	15	82%	43%	97%	82%	43%	97%
