# Supplementary material for: Limitations of the UNAIDS 90-90-90 metrics: a simulation-based comparison of cross-sectional and longitudinal metrics for the HIV care continuum
Source: AIDS. 2020 Feb 14;34(7):1047–55. doi: 10.1097/QAD.0000000000002502 (PMC7253182; doi:10.1097/QAD.0000000000002502)
Supplement: Supplementary file 1 [file aids-34-1047-s002.docx]

# Appendix

Appendix 1: Continuum states over time by repeated cross-section, normal mortality scenario

The stacked area represents the proportion of people in a given state on each day in the simulated scenario. All persons in a given state had passed through all prior states (e.g. persons who are in the “On ART” area had already passed through being at risk for HIV, living with HIV, and Knows status). The death state is shown in the state in which a person was when they died. The vertical bars represent the three simulated periods.

Appendix 2: Continuum states over time by repeated cross-section, high mortality scenario

The stacked area represents the proportion of people in a given state on each day in the simulated scenario. All persons in a given state had passed through all prior states (e.g. persons who are in the “On ART” area had already passed through being at risk for HIV, living with HIV, and knows status). The death state is shown in the state in which a person was when they died. The vertical bars represent the three simulated periods.

Appendix 3: UNAIDS 90-90-90 metrics

|  |  | Baseline mortality scenario |  |  | High mortality scenario |  |  |
| --- | --- | --- | --- | --- | --- | --- | --- |
| Years after simulation start |  | % Knows status out of those who are living with HIV | % On ART out of those who know their status | % Virally suppressed out of those who are on ART | % Knows status out of those who are living with HIV | % On ART out of those who know their status | % Virally suppressed out of those who are on ART |
| Low ART (Period 1) | 1 | 16% | 0% | 33% | 16% | 0% | 33% |
|  | 2 | 29% | 1% | 44% | 29% | 1% | 44% |
|  | 3 | 39% | 2% | 49% | 39% | 2% | 49% |
|  | 4 | 47% | 3% | 61% | 47% | 3% | 61% |
|  | 5 | 53% | 4% | 69% | 53% | 4% | 69% |
| Normal ART (Period 2) | 6 | 59% | 27% | 56% | 59% | 27% | 56% |
|  | 7 | 63% | 40% | 73% | 63% | 40% | 73% |
|  | 8 | 67% | 48% | 81% | 67% | 48% | 81% |
|  | 9 | 70% | 53% | 85% | 70% | 53% | 85% |
|  | 10 | 73% | 58% | 88% | 73% | 58% | 88% |
| Low ART (Period 3) | 11 | 75% | 53% | 96% | 75% | 53% | 96% |
|  | 12 | 77% | 49% | 98% | 77% | 49% | 98% |
|  | 13 | 79% | 46% | 98% | 79% | 46% | 98% |
|  | 14 | 81% | 44% | 98% | 81% | 44% | 98% |
|  | 15 | 82% | 43% | 97% | 82% | 43% | 97% |
